# Lymphangitis carcinomatosa as an unusual presentation of renal cell carcinoma: a case report

**DOI:** 10.1186/1752-1947-2-19

**Published:** 2008-01-24

**Authors:** James E Kirk, Maruti Kumaran

**Affiliations:** 1 Queens Medical Centre, Nottingham, UK

## Abstract

**Introduction:**

Renal cell carcinoma is a common adult malignancy that can present incidentally or with a multitude of clinical symptoms and signs. Metastatic spread is frequent, occurring via haematogenous and lymphatic routes, although it does not typically present with lymphangitis carcinomatosa.

**Case presentation:**

We describe a patient who presented with cough and increasing dyspnoea. Initial chest x-ray and computed tomography were consistent with lymphangitis carcinomatosa that proved secondary to underlying renal cell carcinoma.

**Conclusion:**

Lymphangitis carcinomatosa occurs with many different primary tumours and can rarely be the presenting feature of renal cell carcinoma. Underlying renal cell carcinoma should be considered in the differential diagnosis of lymphangitis carcinomatosa and excluded with subsequent investigations.

## Introduction

Renal cell carcinoma is responsible for 3% of adult malignancies and is the sixth leading cause of cancer death. It is well recognised for its ability to present with atypical symptoms and signs. The classical presentation of loin pain, haematuria and a palpable mass occurs in only around 10% of cases. The list of other recognised presenting symptoms is long and includes pyrexia, hypertension, polycythaemia, left sided varicocoele and distal metastases as well as more non-specific symptoms such as malaise, anorexia and weight loss. However, in current practice more renal cell carcinomas are being detected in asymptomatic patients than from any symptomatic presentation, due to the growing role of imaging in modern healthcare.

Metastatic spread occurs via haematogenous routes and lymphatic routes. Haematogenous metastases occur to almost any part of the body, but particularly to lung, liver and bone as well as involvement of the renal vein and inferior vena cava [1-3]. We describe an unusual case where the initial presentation was precipitated by distant lymphatic spread causing respiratory symptoms.

## Case presentation

A 57-year-old man presented to his general practitioner with symptoms of cough and progressive shortness of breath. He was previously well other than longstanding treated hypertension and he had no significant family history. On further questioning he also described night sweats and unexplained weight loss.

He was referred to the local chest clinic where salient findings on examination included oedema of the extremities, dilated chest wall veins and palpable cervical lymphadenopathy. Examination of the cardiovascular, respiratory and abdominal systems was otherwise unremarkable. Blood tests performed recently had shown elevated ferritin and C reactive protein with otherwise normal haematology and biochemistry results.

**Figure 1:**
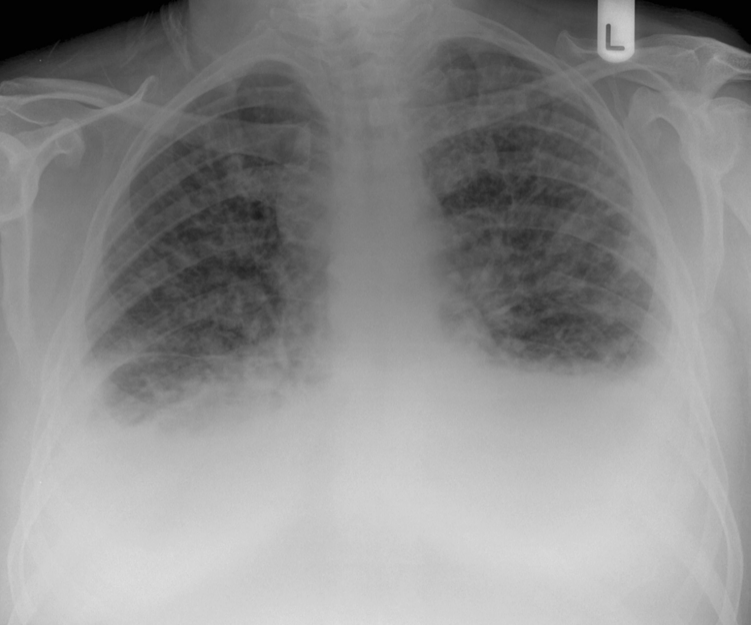
Chest X-ray: bilateral pleural effusions and extensive interstitial reticulo-nodular shadowing.

A chest x-ray performed one month previously at the onset of symptoms was normal. A repeat chest x-ray now showed bilateral pleural effusions and extensive reticulonodular shadowing [Figure 1]. Urgent ultrasound guided biopsy of the neck masses was performed along with CT of the chest, abdomen and pelvis.

Ultrasound examination of the neck confirmed bilateral enlarged lymph nodes in the supraclavicular, and lower and midjugular regions with thrombosis of the right internal jugular vein. Needle biopsy of the lymph nodes was performed.

**Figure 2:**
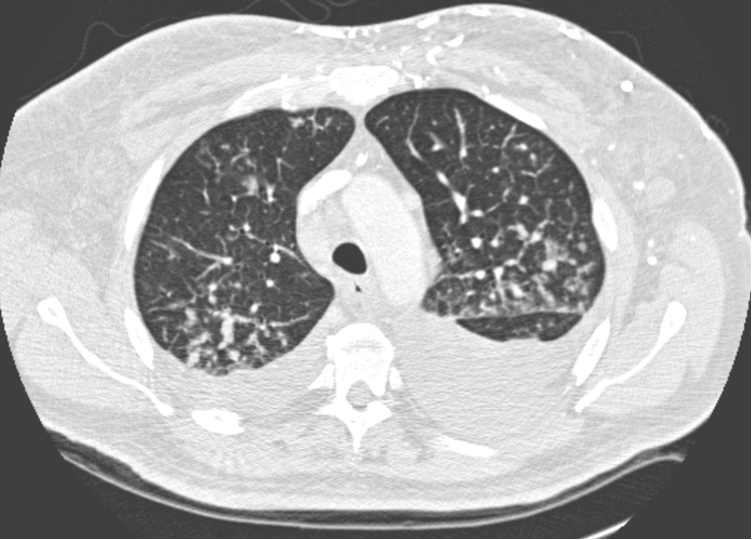
Axial CT of the mid thorax with lung window settings: bilateral pleural effusions and diffuse septal thickening consistent with lymphangitis carcinomatosa.

**Figure 3:**
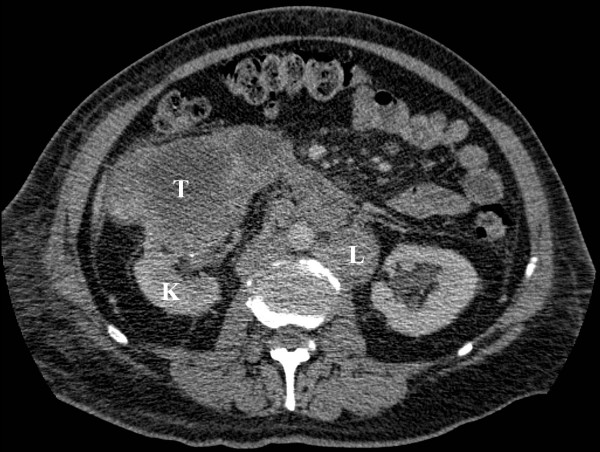
Axial CT through the mid-abdomen following intravenous contrast: the normal part of the right kidney(K) is seen posteriorly with a large mass containing central necrosis arising anteriorly(T). Enlarged lymph nodes(L) are also seen adjacent to the aorta.

CT demonstrated lymphadenopathy affecting the cervical, mediastinal and hilar regions and thrombosis of both jugular veins with extensive collateral circulation. Throughout both lungs there was extensive septal thickening typical of lymphangitis carcinomatosa as well as nodular areas suggestive of pulmonary metastases and moderate bilateral pleural effusions [Figure 2]. Within the abdomen, a 9 × 13 cm mass containing central necrosis was present arising from the lower pole of the right kidney and associated with extensive retroperitoneal lymphadenopathy [Figure 3].

Appearances were those of a renal cell carcinoma (Clinical TNM Stage T3N2M1) with lymphangitis carcinomatosa as part of extensive lymphatic spread. The initial needle biopsy of the cervical lymphadenopathy was inconclusive but a further excision biopsy confirmed the diagnosis of metastatic renal cell carcinoma. Oral steroids and analgesia were commenced but unfortunately the patient passed away within two weeks of the diagnosis.

## Discussion

This case is unusual in that the patient's underlying renal cell carcinoma presented as a result of distant lymphatic spread, primarily due to lymphangitis carcinomatosa. Renal cell carcinomas frequently have regional lymphatic spread at diagnosis, particularly the para-aortic and para-caval nodes, with staging consequently involving careful assessment of potential lymphatic involvement. However, tumours typically present with other features, such as those described in the introduction, before distant lymphatic involvement occurs.

Distant lymph node involvement can be the first recognised feature of renal cell carcinoma and there have been reports of isolated lymph node metastases being detected before a primary renal tumour was apparent [4-7].

Metastatic renal cell carcinoma more frequently affects the lungs via haematogenous spread causing multiple "cannon-ball" metastases but in this case lung involvement was primarily lymphatic.

Lymphangitis carcinomatosa causes dyspnoea and cough, and symptoms often precede chest x-ray findings, as in this case. Appearances on a plain chest radiograph include reticular densities, coarsened bronchovascular markings, Kerley A and B lines, small lung volumes and hilar lymphadenopathy but many patients may have a normal radiograph. Radiological appearances can be difficult to distinguish from many other processes that include pulmonary oedema, interstitial lung diseases and sarcoidosis. Detailed clinical examination is essential and further investigations are often required.

High Resolution Computed Tomography (HRCT) is usually performed in suspected cases or to confirm the diagnosis following a chest x-ray. Features are those of thickened, often beaded, interlobular septae, subpleural thickening, pleural effusions and hilar or mediastinal lymphadenopathy. Although HRCT is more sensitive, it is usually not specific and the entire clinical picture should be taken into account in making the diagnosis. In selected cases, where there is diagnostic uncertainty, a histopathological diagnosis can be obtained via transbronchial or percutaneous biopsy.

Lymphangitis carcinomatosa can occur as the result of many primary tumours, most commonly bronchogenic, breast, stomach, thyroid, pancreatic, laryngeal, colonic, prostatic and cervical carcinomas [8]. In patients with a known malignancy and characteristic imaging findings, the diagnosis is usually straightforward. However in patients who present with clinical and imaging findings consistent with lymphangitis carcinomatosa but no known primary, further investigation and management is guided by thorough review of the patient's history and examination. Depending on clinical findings, further investigations may include bronchoscopy, upper and/or lower gastrointestinal tract endoscopy, mammography, ultrasound or CT. We would advocate the early consideration of CT of the chest and/or abdomen and/or pelvis in patients without an obvious primary. Such an approach would detect many of the potential underlying neoplasms, including renal cell carcinoma, as well as identifying further metastatic disease.

## Conclusion

It is important to remember the wide variety of guises under which renal cell carcinoma may present. Lymphangitis carcinomatosa is recognised to occur as a result of many primary malignancies including renal cell carcinoma. An underlying renal tumour should be considered in cases of distal lymph node metastases or lymphangitis carcinomatosa.

## Abbreviations

CT: computed tomography. HRCT: high resolution computed tomography.

## Competing interests

The author(s) declare that they have no competing interests.

## Authors' contributions

JK: drafted, read and approved the manuscript. MK: read and approved the manuscript.

## Consent

Written informed consent could not be obtained in this case since the patient is deceased and next of kin were untraceable. We believe this case report contains a worthwhile clinical lesson which could not be as effectively made in any other way. We expect the next of kin not to object to the publication since details of the patient remain anonymous.
